# Fear of COVID-19 associated with burnout syndrome in dentists of the Health Directorate of the National Police of Peru: a cross-sectional study at national level under multivariable regression model

**DOI:** 10.1186/s12889-024-18979-9

**Published:** 2024-06-03

**Authors:** Arturo Verástegui-Sandoval, Flor Aquiles-Barzola, Heriberto Machco-Pasmiño, Marysela Ladera-Castañeda, Gissela Briceño-Vergel, Miriam Castro-Rojas, Alberto Cornejo-Pinto, Luis Cervantes-Ganoza, César Cayo-Rojas

**Affiliations:** 1 Research Group “Salud Pública – Salud Integral”, Postgraduate School, Universidad Nacional Federico Villarreal, Lima, Peru; 2 Professional Academic School of Dentistry, Faculty of Health Sciences, Universidad Privada Norbert Wiener, Lima, Peru; 3 School of Stomatology, Universidad Privada San Juan Bautista, Lima, Peru; 4 Faculty of Stomatology, Universidad Inca Garcilaso de La Vega, Lima, Peru

**Keywords:** Burnout syndrome, Fear of Covid-19, Emotional exhaustion, Depersonalization, Self-fulfilment, Peruvian National Police

## Abstract

**Background:**

During the pandemic, many police dentists had the crucial responsibility of ensuring law and order while providing dental care by taking government-approved health measures to stop the spread of the coronavirus. The aim of this study was to assess the association between the fear of COVID-19 and Burnout syndrome in Peruvian dentists belonging to the Health Department of the National Police of Peru (PNP), taking into account possible confounding variables.

**Methods:**

This cross-sectional and analytical study included 182 PNP dentists. The Fear COVID-19 Scale assessed fear of COVID-19 and the Maslach Burnout Inventory Test assessed burnout syndrome. The association between the fear of COVID-19 and Burnout syndrome (self-fulfilment) was analyzed using Spearman's Rho. A multivariable Poisson regression model with a robust variance estimation method was employed to evaluate the impact of fear of COVID-19 on the various dimensions of Burnout syndrome, considering possible confounding variables. The statistical significance level was set at *p* < 0.05.

**Results:**

Under bivariate analysis, fear of COVID-19 was significantly linked with low direct intensity toward emotional exhaustion (Rho = 0.325, *p* < 0.001), very low direct intensity toward depersonalization (Rho = 0.180, *p* = 0.015), and very low inverse intensity toward self-fulfilment (Rho =—0.186, *p* = 0.012). Under multivariable analysis, it was observed that dentists who exhibited fear of COVID-19 were 3.4 and 3.7 times more likely to experience emotional exhaustion and depersonalization, respectively (APR = 3.40, 95% CI: 1.74—6.63 and APR = 3.68, 95% CI: 1.31—10.37), as compared to those who did not display fear of COVID-19. Moreover, none of the potential confounding factors were found to have a significant impact on emotional exhaustion (*p* > 0.05), depersonalization (*p* > 0.05), and self-fulfilment (*p* > 0.05).

**Conclusion:**

Fear of COVID-19 was significantly associated with emotional exhaustion and depersonalization, and inversely associated with self-fulfilment. PNP dentists who exhibited fear of COVID-19 were at greater risk for emotional exhaustion and depersonalization. In developing Burnout syndrome, no significant impact was observed from factors such as age, gender, marital status, children, hierarchy, years of service, work area, private practice, work over 40 h per week, type of service, work performed, sport practice and daily exercise time.

## Background

The COVID-19 pandemic significantly impacted the mental well-being of populations due to the need to implement health measures such as social distancing and limited access to medical and psychological resources to control the spread of the disease, leading to feelings of isolation and loneliness [1]. Additionally, material and economic scarcities were exacerbated, requiring adaptation to teleworking and remote learning [2]. Furthermore, the infodemic spread by certain media outlets has contributed to the increased prevalence of psychological disorders [3].

Dental professionals have been placed in the "very high exposure risk" category by the Occupational Safety and Health Administration (OSHA) due to their exposure to known or suspected sources of the SARS-CoV-2 virus during specific procedures that generate contaminated bioaerosols [4–6]. Due to this, many dentists limited their professional services to emergency or urgent care in accordance with national guidelines [5].

During the pandemic, both healthcare personnel and members of the police and military were exposed to COVID-19 and joined the frontlines in the battle against the virus [7]. Police officers carried the crucial responsibility of ensuring public order and compliance with government-approved sanitary measures to halt the spread of the coronavirus [8–10]. This situation led to an increase in the number of police officers infected with the coronavirus as they came into contact with potentially infected individuals. This increases the risk of the virus spreading to their families and the PNP healthcare workers who exclusively care for them [7, 9, 11].

On 16 May 2020, a Health Directive was approved in Peru to establish dental procedures and biosecurity measures to reduce the risks of COVID-19 transmission in health facilities nationwide [12]. This aspect also included PNP dentists who did not have comorbidity, as they were required to provide dental care to police officers and their immediate family members. Furthermore, certain PNP dentists were directed towards additional duties such as diagnosis, promotion, health prevention, and administrative activities as per the requirements of the service in various health facilities linked to the Police Health Directorate [12].

By March 2023, Peru had reported 4,487,553 cases of infection and 219,539 deaths [13]. During this time, various COVID-19 variants emerged, including XBB, which resulted from the genetic recombination of two Omicron variants [14]. Likewise, the Ministry of the Interior reported that over 520 PNP officers died due to COVID-19, while around 39,000 contracted the disease during the health emergency [10]. Consequently, even after the health emergency, mandatory measures were issued to maintain surveillance, prevention, and control of workers' health at risk of exposure to SARS-CoV-2 in all national health facilities [15].

Concurrent with the risk of infection faced by PNP dentists, they experienced substantial workloads, inadequate resources, extended work hours, sleep disruptions, and a lack of work-life balance. These factors, along with their risk of COVID-19 infection, may have contributed to adverse psychological outcomes such as post-traumatic stress disorder, insomnia, anxiety, depression, fear of COVID-19, and burnout syndrome [10, 16, 17]. Burnout Syndrome is a psychological syndrome that develops as a negative reaction to work stressors, composed of a combination of emotional exhaustion, depersonalization and low self-fulfilment [16, 18]. Emotional exhaustion is linked to an individual's stress experience, which results in a reduction in emotional and physical resources. Depersonalization, or cynicism, results in work detachment as a response to burnout overload, and a loss of enthusiasm and passion for work. The lack of self-fulfilment is characterized by feelings of low professional efficacy and a lack of productivity in personal work [16, 18]. Considering their position within the police force, PNP dentists were likely exposed to work and organizational stressors [19], which may have had an impact on the quality of their professional service and the overall well-being of their institution [16].

Therefore, the aim of this study was to assess the association between the fear of COVID-19 and Burnout syndrome in Peruvian dentists belonging to the Health Department of the National Police of Peru (PNP), taking into account possible confounding variables. Considering as a null hypothesis that there is no association between fear of COVID-19 and Burnout syndrome in Peruvian dentists belonging to the Health Department of the PNP.

## Methods

### Study design

This cross-sectional analytical study was conducted on the entire population of dentists of the Health Directorate of the National Police of Peru between February and May 2023, according to the STROBE guidelines [20].

### Population and selection of participants

The study population consisted of a total of 190 dentists belonging to the Health Department of the National Police of Peru. As we studied the complete populace on a national scale, we did not require a sample size estimate. The ultimate participant count was 182 PNP dentists (*N* = 182), selected based on the following eligibility criteria:

**Inclusion criteria**

- PNP dentists who provided informed consent to voluntarily participate in the study.
- PNP dentists currently engaged in active duty involving assistance and/or administrative responsibilities.

**Exclusion criteria**

- PNP dentists who did not complete the questionnaire.

### Variables

Burnout syndrome in its dimensions of emotional exhaustion, depersonalization and self-fulfilment was considered as a dependent variable. Fear of COVID-19 was considered as an independent variable. Possible confounding variables were age [21, 22], gender [23], marital status [24], number of children [25], hierarchy [22], years of service [23], work area [26], private practice [27], work over 40 h per week [28], type of service [22, 26], work performed [16], sport practice [29] and daily exercise time [30]. The cut-off point for age was based on the median, and with regard to years of service, this cut-off point was based on the number of years a police officer needs as a minimum to be able to apply for retirement without financial loss to himself or to the state. Only if he retires after 20 years of service is he entitled to a permanent pension.

### Application of the instrument

To evaluate the level of fear of COVID-19 among participants, the "Fear COVID-19 Scale (FCV-19S)" was utilized. The scale is composed of 7 items with responses rated on a Likert scale ranging from 1 (strongly disagree) to 5 (strongly agree). The total scores ranged from 7 to 35 and individuals with scores between 17 and 35 are identified as having fear of COVID-19 [31, 32]. The reliability of the scale was evaluated using Cronbach's alpha, obtaining a value of α = 0.884 (95% CI: 0.856—0.908), which is considered acceptable.

The study employed the Maslach Burnout Inventory Test to evaluate Burnout syndrome, which included 22 questions grouped into three dimensions. The emotional exhaustion dimension had nine items (Q1, Q2, Q3, Q6, Q8, Q13, Q14, Q16, and Q20), depersonalization had five items (Q5, Q10, Q11, Q15, and Q22), and self-fulfilment had eight items (Q4, Q7, Q9, Q12, Q17, Q18, Q19, and Q21). Responses were evaluated on a Likert scale ranging from 0 to 6, with 0 indicating never, 1 indicating once a year, 2 indicating once a month, 3 indicating sometimes a month, 4 indicating once a week, 5 indicating sometimes a week, and 6 indicating every day. The ratings for the dimensions of Burnout Syndrome were as follows: Emotional exhaustion ratings ranging from 0 to 18 indicating low or mild levels, from 19 to 26 indicating medium or moderate levels, and from 27 to 54 indicating high or severe levels; Depersonalization ratings ranging from 0 to 5 indicating low levels, from 6 to 9 indicating medium levels, and from 10 to 30 indicating high levels. For self-fulfilment, scores from 40 to 48 were considered low, 34 to 39 were considered medium, and 0 to 33 were considered high [33]. Indications of burnout included a total score of more than 26 points for emotional exhaustion, more than 9 points for depersonalization, and less than 34 points for self-fulfilment [34]. The Cronbach's alpha coefficient was used to determine the internal consistency of the instrument. The result was α = 0.780 (95% CI: 0.731—0.824), which is considered acceptable.

### Procedure

The survey was conducted using the Google Forms® platform and distributed to the institutional or personal email address of every dentist in a self-administered manner. If there was no response, the invitation was re-shared to their personal email or WhatsApp®, up to three times within a three-week period. The principal investigator (A.V.S) provided the invitation. Upon clicking the provided web link, dentists were promptly directed to the informed consent, inclusive of the institutional email, phone, and full name of the principal investigator. Participants were also afforded access to ethics committee data. If they chose to consent, they were automatically redirected to the next page, which contained the Questionnaire and accompanying instructions. Participants had the right to decline the invitation or choose not to complete the questionnaire. The data were accessible only to the principal investigator and were securely stored on a digital device to ensure the confidentiality of the data throughout the entire survey process. Only one complete response per dentist was accepted and to prevent duplication, only one response per associated email was allowed. Additionally, participants were requested to enter the initials of their first and last names, along with their age (for instance: AVS56). This was done to avoid duplications in case someone accessed the web link from multiple email addresses. The participants were not given any incentives for their involvement and had access to the link from February 15 through to May 20, 2023.

### Data analysis

The statistical calculations were conducted using IBM's Statistical Package for the Social Sciences (SPSS) version 28.0 (IBM, Armonk, New York, USA). The qualitative variables' statistical description utilized absolute and relative frequencies, while the quantitative variables' measures of central tendency and dispersion relied on mean and standard deviation. To assess Burnout Syndrome's three dimensions' correlation with fear of COVID-19, Spearman's Rho was the statistical test employed. Additionally, we utilized Pearson's Chi-square test for the bivariate analysis of categorical variables and Fisher's exact test for expected values less than 5. For the multivariable analysis, we employed a multivariable Poisson regression model with robust variance to assess the impact of fear of COVID-19 on the three dimensions of Burnout syndrome, while considering possible confounding variables. All analyses were conducted with a significance level of *p* < 0.05.

### Ethical aspects

The present study adhered to the bioethical principles outlined in the Declaration of Helsinki related to respect, freedom, nonmaleficence, and confidentiality [35]. Approval was obtained from the Ethics Committee of the Postgraduate School at the Universidad Nacional Federico Villarreal with letter No. 013–2023-UIIE-EUPG-UNFV dated February 13, 2023. Furthermore, participants were requested to provide their voluntary informed consent on the initial page of the online survey.

## Results

The dentist response rate for the PNP Health Directorate was 95.8% of the total population, with a mean age of 37.3 ± 10.5 years. Of this group, 52.7% were ≥ 35 years old, 54.4% were female, and 51.6% were single and childless. Within the population, 72.5% held junior officer positions and 76.4% had ≤ 20 years of service. 77.5% of dentists lived in the Peruvian capital, with 58.8% also practicing privately. Seventy-two percent of the personnel worked more than 40 h weekly, with 59.3% employed in the general dental division of the police. Additionally, 39% of all personnel worked in police administrative or support roles. Finally, 64.3% of all personnel engaged in sport practice and 45.1% exercised for no more than 30 min each day (Table 1).

**Table 1:** Sociodemographic characteristics of dentists of the National Police of Peru (PNP)

**Variable**	**Category**	**Frequency**	**Percentage**
**Age group**	< 35 years	86	47.3
	≥ 35 years	96	52.7
**Gender**	Male	83	45.6
	Female	99	54.4
**Marital status**	Married or cohabiting	88	48.4
	Single	94	51.6
**Children**	Without children	94	51.6
	1 child	46	25.3
	≥ 2 children	42	23.1
**Hierarchy**	Junior Officer	132	72.5
	Senior Officer	50	27.5
**Years of service**	> 20 years	43	23.6
	≤ 20 years	139	76.4
**Work area**	Province	41	22.5
	Capital	141	77.5
**Private practice**	No	75	41.2
	Yes	107	58.8
**Works more than 40 h per week**	No	51	28.0
	Yes	131	72.0
**Type of service**	General Dentistry	108	59.3
	Specialty	74	40.7
**Work performed**	Management and assistance	33	18.1
	Assistance	59	32.4
	Administrative	19	10.4
	Assistance and administrative	71	39.0
**Sport practice**	No	65	35.7
	Yes	117	64.3
**Daily exercise time**	None	47	25.8
	≤ 30 min	82	45.1
	> 30 min	53	29.1
**Age**	**Mean**	**Median**	**SD**
	37.3	35.0	10.5

*SD:* Standard deviation

**Fig. 1:**
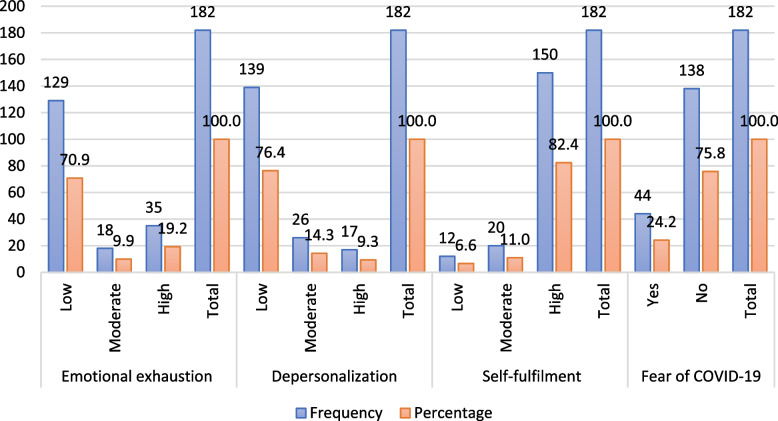
Frequency of Burnout Syndrome (according to dimensions of emotional exhaustion, depersonalization and self-fulfilment) and fear of COVID-19 in dentists of the PNP Health Directorate

Of the 182 participants, 19.2% (95% CI: 13.5%—25.0%) exhibited burnout syndrome related to emotional exhaustion (high), 9.3% (95% CI: 5.1%—13.6%) related to depersonalization (high), and 6.6% (95% CI: 3.0%—10.2%) related to self-fulfilment (low). Additionally, 24.2% (95% CI: 18.0%—30.4%) of participants reported fear of COVID-19 (Fig. 1).

Fear of COVID-19 was found to be significantly linked to low direct intensity to emotional exhaustion (Rho = 0.325, *p* < 0.001), very low direct intensity to depersonalization (Rho = 0.180, *p* = 0.015), and very low inverse intensity to self-fulfilment (Rho = -0.186, *p* = 0.012), according to the obtained score correlation (Table 2).

**Table 2:** Correlation between fear of COVID-19 score and emotional exhaustion, depersonalization and self-fulfilment scores

**Correlation of scores**		**Spearman's Rho**	**95% CI**		*p
			**LL**	**UL**	
Fear of COVID-19	Emotional exhaustion	0.325	0.184	0.452	<0.001*
	Depersonalization	0.180	0.031	0.321	0.015*
	Self-fulfilment	-0.186	-0.326	-0.037	0.012*

^*^Based on Spearman's Rho (*p* < 0.05, significant correlation)

The bivariate analysis indicates a significant association between fear of COVID-19 and age group, marital status, children, hierarchy, years of service, and work performed (*p* < 0.001, *p* = 0.020, *p* < 0.001, *p* < 0.001, *p* < 0.001, *p* < 0.001, and *p* = 0.002, respectively). Additionally, there is a significant association between the level of emotional exhaustion and hierarchy, years of service, and work area (*p* = 0.006, *p* = 0.043, and *p* = 0.030, respectively). There were significant associations found between gender, marital status, and work area with the level of self-fulfilment (*p* = 0.046, *p* = 0.029, and *p* < 0.001, respectively). Finally, the level of depersonalization was not significantly associated with any of the sociodemographic variables considered in this study (*p* > 0.05) (Table 3).

**Table 3:** Levels of Fear of COVID-19, emotional exhaustion, depersonalization and self-fulfilment associated with sociodemographic variables of PNP dentists

Variable	Category	Fear of COVID-19			Emotional exhaustion				Depersonalization				Self-fulfilment			
		Yes	No	*p*	Low	Moderate	High	*p*	Low	Moderate	High	*p*	Low	Moderate	High	*p*
		f (%)	f (%)		f (%)	f (%)	f (%)		f (%)	f (%)	f (%)		f (%)	f (%)	f (%)	
**Age group**	< 35 years	10 (11.6)	76 (88.4)	< 0.001*	66 (76.7)	8 (9.3)	12 (14.0)	0.201	63 (73.3)	13 (15.1)	10 (11.6)	0.549	5 (5.8)	6 (7.0)	75 (87.2)	0.224
	≥ 35 years	34 (35.4)	62 (64.6)		63 (65.6)	10 (10.4)	23 (24.0)		76 (79.2)	13 (13.5)	7 (7.3)		7 (7.3)	14 (14.6)	75 (78.1)	
**Gender**	Male	22 (26.5)	61 (73.5)	0.501	56 (67.5)	9 (10.8)	18 (21.7)	0.648	62 (74.7)	11 (13.3)	10 (12.0)	0.505	9 (10.8)	6 (7.2)	68 (81.9)	0.046*
	Female	22 (22.2)	77 (77.8)		73 (73.7)	9 (9.1)	17 (17.2)		77 (77.8)	15 (15.2)	7 (7.1)		3 (3.0)	14 (14.1)	82 (82.8)	
**Marital status**	Married or cohabiting	28 (31.8)	60 (68.2)	0.020*	60 (68.2)	8 (9.1)	20 (22.7)	0.505	69 (78.4)	12 (13.6)	7 (8.0)	0.781	4 (4.5)	15 (17.0)	69 (78.4)	0.029*
	Single	16 (17.0)	78 (83.0)		69 (73.4)	10 (10.6)	15 (16.0)		70 (74.5)	14 (14.9)	10 (10.6)		8 (8.5)	5 (5.3)	81 (86.2)	
**Children**	Without children	14 (14.9)	80 (85.1)	< 0.001*	66 (70.2)	12 (12.8)	16 (17.0)	0.705	69 (73.4)	12 (12.8)	13 (13.8)	0.237	9 (9.6)	11 (11.7)	74 (78.7)	0.473
	1 child	11 (23.9)	35 (76.1)		34 (73.9)	3 (6.5)	9 (19.6)		37 (80.4)	8 (17.4)	1 (2.2)		2 (4.3)	6 (13.0)	38 (82.6)	
	≥ 2 children	19 (45.2)	23 (54.8)		29 (69.0)	3 (7.1)	10 (23.8)		33 (78.6)	6 (14.3)	3 (7.1)		1 (2.4)	3 (7.1)	38 (90.5)	
**Hierarchy**	Junior officer	21 (15.9)	111 (84.1)	< 0.001*	102 (77.3)	9 (6.8)	21 (15.9)	0.006*	98 (74.2)	19 (14.4)	15 (11.4)	0.302	7 (5.3)	14 (10.6)	111 (84.1)	0.487
	Senior officer	23 (46.0)	27 (54.0)		27 (54.0)	9 (18.0)	14 (28.0)		41 (82.0)	7 (14.0)	2 (4.0)		5 (10.0)	6 (12.0)	39 (78.0)	
**Years of service**	> 20 years	22 (51.2)	21 (48.8)	< 0.001*	24 (55.8)	6 (14.0)	13 (30.2)	0.043*	35 (81.4)	6 (14.0)	2 (4.7)	0.469	2 (4.7)	6 (14.0)	35 (81.4)	0.738
	≤ 20 years	22 (15.8)	117 (84.2)		105 (75.5)	12 (8.6)	22 (15.8)		104 (74.8)	20 (14.4)	15 (10.8)		10 (7.2)	14 (10.1)	115 (82.7)	
**Work area**	Province	9 (22.0)	32 (78.0)	0.705	29 (70.7)	6 (14.6)	6 (14.6)	0.409	30 (73.2)	9 (22.0)	2 (4.9)	0.185	9 (22.0)	5 (12.2)	27 (65.9)	< 0.001**
	Capital	35 (24.8)	106 (75.2)		100 (70.9)	12 (8.5)	29 (20.6)		109 (77.3)	17 (12.1)	15 (10.6)		3 (2.1)	15 (10.6)	123 (87.2)	
**Private practice**	No	17 (22.7)	58 (77.3)	0.691	55 (73.3)	6 (8.0)	14 (18.7)	0.744	56 (74.7)	12 (16.0)	7 (9.3)	0.856	7 (9.3)	12 (16.0)	56 (74.7)	0.071
	Yes	27 (25.2)	80 (74.8)		74 (69.2)	12 (11.2)	21 (19.6)		83 (77.6)	14 (13.1)	10 (9.3)		5 (4.7)	8 (7.5)	94 (87.9)	
**Works more than 40 h per week**	No	13 (25.5)	38 (74.5)	0.796	40 (78.4)	3 (5.9)	8 (15.7)	0.336	40 (78.4)	7 (13.7)	4 (7.8)	0.894	4 (7.8)	8 (15.7)	39 (76.5)	0.388
	Yes	31 (23.7)	100 (76.3)		89 (67.9)	15 (11.5)	27 (20.6)		99 (75.6)	19 (14.5)	13 (9.9)		8 (6.1)	12 (9.2)	111 (84.7)	
**Type of service**	General dentistry	28 (25.9)	80 (74.1)	0.505	77 (71.3)	10 (9.3)	21 (19.4)	0.942	82 (75.9)	15 (13.9)	11 (10.2)	0.887	8 (7.4)	15 (13.9)	85 (78.7)	0.254
	Specialty	16 (21.6)	58 (78.4)		52 (70.3)	8 (10.8)	14 (18.9)		57 (77.0)	11 (14.9)	6 (8.1)		4 (5.4)	5 (6.8)	65 (87.8)	
**Work performed**	Management and assistance	7 (21.2)	26 (78.8)	0.002*	21 (63.6)	4 (12.1)	8 (24.2)	0.030**	27 (81.8)	3 (9.1)	3 (9.1)	0.735	1 (3.0)	2 (6.1)	30 (90.9)	0.721
	Assistance	9 (15.3)	50 (84.7)		44 (74.6)	3 (5.1)	12 (20.3)		43 (72.9)	8 (13.6)	8 (13.6)		5 (8.5)	6 (10.2)	48 (81.4)	
	Administrative	11 (57.9)	8 (42.1)		8 (42.1)	4 (21.1)	7 (36.8)		14 (73.7)	3 (15.8)	2 (10.5)		0 (0.0)	3 (15.8)	16 (84.2)	
	Assistance and administrative	17 (23.9)	54 (76.1)		56 (78.9)	7 (9.9)	8 (11.3)		55 (77.5)	12 (16.9)	4 (5.6)		6 (8.5)	9 (12.7)	56 (78.9)	
**Sport practice**	No	19 (29.2)	46 (70.8)	0.235	40 (61.5)	10 (15.4)	15 (23.1)	0.078	50 (76.9)	10 (15.4)	5 (7.7)	0.825	3 (4.6)	8 (12.3)	54 (83.1)	0.682
	Yes	25 (21.4)	92 (78.6)		89 (76.1)	8 (6.8)	20 (17.1)		89 (76.1)	16 (13.7)	12 (10.3)		9 (7.7)	12 (10.3)	96 (82.1)	
**Daily exercise time**	None	13 (27.7)	34 (72.3)	0.084	28 (59.6)	8 (17.0)	11 (23.4)	0.214	37 (78.7)	7 (14.9)	3 (6.4)	0.845	1 (2.1)	4 (8.5)	42 (89.4)	0.167
	≤ 30 min	24 (29.3)	58 (70.7)		59 (72.0)	7 (8.5)	16 (19.5)		64 (78.0)	10 (12.2)	8 (9.8)		5 (6.1)	7 (8.5)	70 (85.4)	
	> 30 min	7 (13.2)	46 (86.8)		42 (79.2)	3 (5.7)	8 (15.1)		38 (71.7)	9 (17.0)	6 (11.3)		6 (11.3)	9 (17.0)	38 (71.7)	

^*^Based on Pearson's chi-square (*p < 0.05, significant association). For expected values less than 5, Fisher's exact test was used (**p < 0.05, significant association)

In the Poisson regression analysis with robust variance, using Prevalence Ratio (PR), Burnout was considered positive for emotional exhaustion (Yes [> 26 points] = 1 / No [≤ 26 points] = 0), depersonalization (Yes [> 9 points] = 1 / No [≤ 9 points] = 0), and lack of self-fulfilment (Yes [< 34 points] = 1 / No [≥ 34 points] = 0). These three variables were studied as the dependent variables, while Fear of COVID-19 (Yes = 1 / No = 0) was studied as the independent variable. Age group, sex, marital status, children, hierarchy, years of service, work area, private practice, working more than 40 h per week, type of service, work performed, sport practice, and daily exercise time were the possible confounding variables. After adjusting the prevalence ratio (APR) of the model, it was found that dentists who displayed fear of COVID-19 were 3.4 and 3.7 times more likely to experience emotional exhaustion and depersonalization (with APRs of 3.40, 95% CI: 1.74–6.63 and 3.68, 95% CI: 1.31–10.37, respectively) as compared to their counterparts who didn't demonstrate COVID-19-related fear. Furthermore, none of the confounding variables were found to be significant factors in emotional exhaustion (*p* > 0.05), depersonalization (*p* > 0.05), and lack of self-fulfilment (*p* < 0.05) (Table 4).

**Table 4:** Adjusted regression analysis model of emotional exhaustion, depersonalization and self-fulfilment associated with Fear of COVID-19 and sociodemographic variables

Variable	Category	Adjusted Prevalence Ratio Model (APR) with robust variance														
		Emotional exhaustion					Depersonalization					Lack of self-fulfilment				
		β	APR	95% CI		p*	β	APR	95% CI		p*	β	APR	95% CI		*p*
				LL	UL				LL	UL				LL	UL	
**Fear of COVID-19**	Yes	1.22	3.40	1.74	6.63	< 0.001*	1.30	3.68	1.31	10.37	0.014*	-0.16	0.85	0.21	3.43	0.817
	No		*Ref*					*Ref*					*Ref*			
**Age group**	< 35 years	-0.19	0.83	0.35	1.98	0.675	0.14	1.15	0.29	4.49	0.844	-0.80	0.45	0.13	1.53	0.201
	≥ 35 years		*Ref*					*Ref*					*Ref*			
**Gender**	Female	-0.15	0.86	0.44	1.67	0.652	-0.93	0.39	0.14	1.13	0.083	-1.22	0.30	0.08	1.17	0.082
	Male		*Ref*					*Ref*					*Ref*			
**Marital status**	Single	-0.37	0.69	0.34	1.40	0.308	-0.47	0.62	0.23	1.68	0.351	0.21	1.24	0.52	2.94	0.632
	Married or cohabiting		*Ref*					*Ref*					*Ref*			
**Children**	Without children	0.75	2.12	0.67	6.75	0.201	0.86	2.36	0.39	14.15	0.347	2.42	11.24	1.05	120.25	0.045
	1 child	0.35	1.42	0.61	3.32	0.417	-1.19	0.30	0.04	2.14	0.232	0.99	2.72	0.22	33.16	0.433
	≥ 2 children		*Ref*					*Ref*					*Ref*			
**Hierarchy**	Senior officer	0.28	1.32	0.23	7.60	0.757	-1.71	0.18	0.03	1.12	0.066	1.24	3.47	0.95	12.69	0.060
	Junior officer		*Ref*					*Ref*					*Ref*			
**Years of service**	≤ 20 years	-0.07	0.93	0.15	5.80	0.936	-0.91	0.40	0.07	2.21	0.294	0.00	1.00	0.09	11.83	0.998
	> 20 years		*Ref*					*Ref*					*Ref*			
**Work area**	Capital	0.08	1.09	0.44	2.68	0.857	0.96	2.60	0.74	9.21	0.138	-3.18	0.04	0.00	0.56	0.017
	Province		*Ref*					*Ref*					*Ref*			
**Private practice**	Yes	-0.14	0.87	0.43	1.76	0.701	0.04	1.04	0.24	4.59	0.957	-1.45	0.24	0.04	1.28	0.094
	No		*Ref*					*Ref*					*Ref*			
**Works more than 40 h per week**	Yes	0.36	1.43	0.60	3.45	0.422	-0.02	0.98	0.19	4.97	0.981	1.79	5.96	0.65	54.42	0.114
	No		*Ref*					*Ref*					*Ref*			
**Type of service**	Specialty	-0.08	0.92	0.42	2.04	0.845	-0.66	0.52	0.19	1.39	0.193	0.60	1.83	0.23	14.56	0.568
	General dentistry		*Ref*					*Ref*					*Ref*			
**Work performed**	Management and assistance	0.81	2.25	0.93	5.49	0.073	0.89	2.43	0.57	10.47	0.232	-1.26	0.28	0.01	5.65	0.409
	Assistance	0.78	2.17	0.99	4.72	0.051	0.95	2.58	0.79	8.35	0.115	2.25	9.48	0.65	137.61	0.099
	Administrative	0.60	1.82	0.71	4.67	0.215	0.52	1.68	0.24	11.85	0.604	*Omitted*				
	Assistance and administrative		*Ref*					*Ref*					*Ref*			
**Sport practice**	Yes	-0.19	0.83	0.39	1.75	0.622	-0.37	0.69	0.14	3.49	0.656	-0.55	0.58	0.13	2.49	0.461
	No		*Ref*					*Ref*					*Ref*			
**Daily exercise time**	None	-0.12	0.89	0.29	2.75	0.839	-1.19	0.31	0.03	2.74	0.289	-0.76	0.47	0.04	5.60	0.547
	≤ 30 min	-0.14	0.87	0.34	2.18	0.759	-0.42	0.66	0.22	1.99	0.459	-0.26	0.77	0.20	2.92	0.704
	> 30 min		*Ref*					*Ref*					*Ref*			
**Constant of the model**		-2.81	0.06	0.01	0.59	0.016	-1.96	0.14	0.00	7.01	0.326	-3.06	0.05	0.00	1.32	0.073

*APR:* Adjusted Prevalence Ratio under the Poisson regression model with robust variance, *β*: Coefficient of determination. *95% CI:* 95% Confidence Interval, *LL:* Lower Limit, *UL:* Upper Limit

^*^Adjusted multiple regression model (*p < 0.05, significant association)

## Discussion

During the COVID-19 pandemic, frontline teams, including health care workers, police, and military personnel, were at a heightened risk of infection due to their direct contact with potentially infected individuals [8, 9]. Dentists, similarly, were exposed to stressors such as working in high-risk environments, inadequate working conditions, and constant concern regarding the availability of personal protective equipment (PPE) to shield them from salivary and blood aerosols [36–38]. This situation could result in significant subjective overload, fear, and psychological distress paired with emotional exhaustion, helplessness, depersonalization, negative attitudes towards work and life, and low self-fulfilment [39–41]. Therefore, the aim of this study was to assess the association between the fear of COVID-19 and Burnout syndrome in Peruvian dentists belonging to the Health Department of the PNP, taking into account possible confounding variables. Therefore, according to the results obtained, the null hypothesis was rejected.

The study's results reveal Burnout syndrome in 19.2% of all dentists surveyed, with high emotional exhaustion observed. Additionally, high depersonalization was identified in 9.3%, and low self-fulfilment in 6.6% of respondents. These findings contrast with those of Silva et al. [41], who discovered that among Brazilian dentists, 43.4% reported high levels of personal exhaustion, 26.3% indicated high levels of depersonalization, and 81.3% reported low levels of self-fulfilment. This may be attributed to the fact that the present study was conducted at the onset of 2023, during the final phase of the COVID-19 public health crisis in Peru, where accurate information was readily available and a significant proportion of the adult populace had already obtained 3 to 4 immunization shots. In addition, it is possible that the participants in the current study exhibited more resilience due to their experience as police dentists handling dangerous situations [2, 23]. This is in contrast to Silva et al.'s 2020 study [41], which was conducted during the second wave of COVID-19 infections when many dentists were exposed to high levels of anxiety, depression, and stress, resulting in an increase in burnout levels [42, 43].

The present study found that 24.2% of respondents were fearful of COVID-19, which differs from Salehiniya et al.'s [44] report indicating 85% of dentists were scared of contracting the virus. Furthermore, our results were incongruent with Birant and Gümüştaş' study [17], which identified that 78% of dentists were afraid of being infected with COVID-19. These discrepancies may have been due to the fact that these studies were conducted between 2020 and 2021, a period in which dentists were affected by the infodemic and the beginning of the implementation of the vaccination process, which created a degree of uncertainty about the efficacy of the vaccines [45–49].

Likewise, our findings indicated that fear of COVID-19 was significantly associated with low direct intensity with emotional exhaustion, with very low direct intensity with depersonalization, and with very low inverse intensity with self-fulfilment. These findings align with those of Ahorsu et al. [50] and Lacerda et al. [51], who observed a positive correlation between COVID-19 fear and emotional exhaustion and depersonalization in healthcare workers, but a negative correlation with self-fulfilment [50, 51]. This suggests that as perceived threat of COVID-19 rises, emotional exhaustion and depersonalization symptoms likewise increase, albeit in low intensities. However, increasing fear of COVID-19 appears to diminish the sense of self-fulfilment also to a low degree. This observation suggests that Peruvian police dentists may possess a certain level of resilience in the midst of life-threatening danger. Additionally, receiving four vaccine doses may have provided a sense of security against infection [50, 52].

The bivariate analysis revealed a significant correlation between fear of COVID-19 and age group, as well as years of service. These findings mirror those of Teutli-Mellado et al. [21], who observed that dentists over 40 years of age experienced greater levels of fear compared to their 20–40-year-old counterparts. This can be attributed to older dentists feeling more susceptible to infection [21]. Fear of COVID-19 was associated with marital status, as married dentists were afraid of infecting their family members during clinical activities and other duties of enforcement [53, 54]. The number of children was also linked to fear of COVID-19, potentially due to a higher risk of cross-infection among family members [4, 54]. Furthermore, fear of COVID-19 was found to be linked to police hierarchy. The heightened fear among police personnel during the pandemic may be attributed to the greater responsibility placed upon higher-ranking official dentists, who oversee multiple work teams within the PNP. Unlike other hazardous situations, the COVID-19 pandemic has exposed all members of the police force to the same perilous circumstances, irrespective of their rank [22]. Finally, fear of COVID-19 was correlated with the type of work performed. The findings contrast with those of Birant and Gümüştaş [17], who observed that dentists in administrative roles exhibited lower levels of fear of infection [17]. The present study identified that dentists in administrative police roles experienced greater fear of contagion than those in custodial roles. In the final stage of the health emergency, masks were not required, but those providing assistance always had personal protective equipment (PPE) when in contact with patients. This may have provided a heightened sense of security [55].

Based on a multivariable regression analysis, dental police officers who feared COVID-19 were 3.4 and 3.7 times more likely to exhibit emotional exhaustion and depersonalization, respectively, compared to those who did not fear the virus. These findings align with those reported by Zambrano et al. [52], who noted that healthcare professionals with high levels of COVID-19 fear experienced burnout. The pandemic posed a threat to dentists, healthcare professionals, and police officers due to their law enforcement responsibilities and changes in their roles prompted by government directives aimed at reducing the spread of the disease [8, 19, 52]. This may have placed a significant emotional burden on them, which could have negative implications for their psychological health. As a result, these professionals may have experienced increased emotional exhaustion and depersonalization [52, 56].

Although some sociodemographic factors were found to be associated with the emotional exhaustion and depersonalization dimensions of Burnout syndrome according to the bivariate analysis, the multivariable regression analysis observed no significant influence of any confounding variable in any of the three dimensions. This demonstrates that a bivariate association does not always signify causality or influence [57–59]. No sociodemographic factor was found to be decisive in the presentation of Burnout characteristics among the respondents. This observation suggests that the fear of COVID-19 experienced by PNP dentists might be a potential risk factor for the development of emotional exhaustion and depersonalization, irrespective of age, gender, family or work conditions, given their arduous and disciplined training to confront critical situations. [22]. However, it should be acknowledged based on the findings that the pandemic did affect their job duties, as the extent of the illness was unforeseen and unprecedented [60, 61]. It must also be acknowledged that the COVID-19 pandemic may have had some negative effects on the mental health and burnout of the workforce, so it is important that police dentists develop resilience, i.e. the personal ability to adapt positively to sudden unexpected events and widespread challenges, as this will enable them to fulfil their duties while protecting their mental health and personal well-being [62–64].

One advantage of this study is that all PNP dentists from all over the country were invited to participate, thus providing new and unprecedented results in Peru, which is very important because dental police officers are frontline risk personnel. They endure psychological pressure while trying to balance care with other PNP responsibilities [12, 53], and many also have familial obligations [53]. The obtained results will aid in developing future protocols for the care and training of police health personnel and preparing for emergencies similar to the pandemic, avoiding uncertainties that could affect their mental health and work performance [65].

Limitations of this study include the inability to compare dentists in other sectors, such as the Ministry of Health (MINSA), Social Security, and private entities. Another limitation was that the pre-existence of mental or chronic illnesses was not taken into account, so it is suggested that these be included in future studies, as these factors may have contributed to burnout [66]. Additionally, this cross-sectional research did not allow for the evaluation of the dynamics and sustainability of Burnout syndrome in PNP dentists during and after the pandemic. It is recommended that health and government authorities should conduct regular mental health checkups, provide counseling and psychological support, and offer coping and resilience strategies for PNP dentists to detect any potential changes that may impact their mental health and work productivity [67, 68]. Additionally, longitudinal studies should be conducted to further explore this research topic in the post-pandemic era.

## Conclusion

Fear of COVID-19 was significantly associated with emotional exhaustion and depersonalization, and inversely associated with self-fulfilment. PNP dentists who were afraid of COVID-19 were at greater risk for emotional exhaustion and depersonalization. Factors such as age, sex, marital status, children, hierarchy, years of service, work zone, private practice, working over 40 h per week, type of service, work performed, sport practice and daily exercise time were not considered to be influential factors in the development of burnout syndrome.

## Data Availability

All data analyzed during this study are available from the corresponding author on reasonable request (cesarcayorojas@gmail.com).

## Abbreviations

APR: Adjusted Prevalence Ratio
CI: Confidence interval
COVID-19: COronavirus DIsease 2019
OSHA: Occupational Safety and Health Administration
PNP: National Police of Peru
STROBE: STrengthening the Reporting of OBservational studies in Epidemiology
SPSS: Statistical Package for the Social Sciences
